# Associations between Plasma Biomarkers and Cognition in Patients with Alzheimer’s Disease and Amnestic Mild Cognitive Impairment: A Cross-Sectional and Longitudinal Study

**DOI:** 10.3390/jcm8111893

**Published:** 2019-11-06

**Authors:** Chia-Lin Tsai, Chih-Sung Liang, Jiunn-Tay Lee, Ming-Wei Su, Chun-Chieh Lin, Hsuan-Te Chu, Chia-Kuang Tsai, Guan-Yu Lin, Yu-Kai Lin, Fu-Chi Yang

**Affiliations:** 1 Department of Neurology, Tri-Service General Hospital, National Defense Medical Center, Taipei 114, Taiwan; 2 Department of Psychiatry, Beitou Branch, Tri-Service General Hospital, National Defense Medical Center, Taipei 112, Taiwan; 3 Institute of Biomedical Sciences, Academia Sinica, Taipei 115, Taiwan

**Keywords:** Alzheimer’s disease, amnestic mild cognitive impairment, plasma biomarkers, immunomagnetic reduction

## Abstract

Brain degeneration in patients with Alzheimer’s disease (AD) results from the accumulation of pathological amyloid-β (Aβ) plaques and tau protein tangles, leading to altered plasma levels of biomarkers. However, few studies have investigated the association between plasma biomarkers and cognitive impairment in patients with AD. In this cross-sectional study, we investigated correlations between mini-mental state examination (MMSE) scores and levels of plasma biomarkers in patients with amnestic mild cognitive impairment (aMCI) and AD. Thirteen individuals with normal cognition, 40 patients with aMCI, and 37 patients with AD were enrolled. Immunomagnetic reduction was used to assess the levels of plasma biomarkers, including amyloid Aβ_1-40_, Aβ_1-42_, total tau protein (t-Tau), and phosphorylated tau protein (threonine 181, p-Tau181). Our analysis revealed a significant negative correlation between MMSE and both measures of tau, and a trend toward negative correlation between MMSE and Aβ_1-42_. In a longitudinal study involving three patients with aMCI and two patients with AD, we observed strong negative correlations (r < −0.8) between changes in MMSE scores and plasma levels of t-Tau. Our results suggest that plasma levels of t-Tau and p-Tau181 can be used to assess the severity of cognitive impairment in patients with AD. Furthermore, the results of our preliminary longitudinal study suggest that levels of t-Tau can be used to monitor the progression of cognitive decline in patients with aMCI/AD.

## 1. Introduction

Patients with cognitive impairment exhibit decreased abilities across several cognitive domains, including attention, calculation, recall, language, orientation, and the ability to follow simple commands [1,2,3]. In Alzheimer’s disease (AD) patients, brain degeneration is believed to result from the accumulation of amyloid-β (Aβ) plaques and tau protein tangles in the brain [4,5,6]. Such accumulation leads to neuronal damage, and in turn to hippocampal atrophy, cortical thinning, and brain dysfunction [7,8,9].

As cognitive impairment develops in AD patients, concentrations of AD-related biomarkers in body fluids are altered due to the formation of Aβ plaques and tau protein in the brain [10,11,12]. Previous studies have reported decreased cerebrospinal fluid (CSF) Aβ_1-42_ and increased CSF total tau (t-Tau) levels in AD, as detected by an enzyme-linked immunosorbent assay (ELISA) and other conventional immunoassays [13,14,15]. Thus, CSF levels of Aβ_1-42_ and t-Tau are, respectively, positively and negatively correlated with scores on the mini-mental state examination (MMSE), which is used to assess the degree of cognitive impairment in patients with dementia (defined as score < 26). Several studies have also shown that plasma t-Tau levels are higher in AD patients than in healthy subjects [16,17]. Although most investigations reported no differences in plasma Aβ_1-42_ levels, independent studies based on immunomagnetic reduction (IMR) reported increased plasma Aβ_1-42_ levels in AD. IMR assays exhibited better consistence and sensitivity in the quantification of extremely low concentrations of plasma Aβ_1-42_ from diverse and independent cohorts, compared with conventional ELISA. Further studies confirmed opposite changes in Aβ_1-42_ levels in the plasma and CSF of AD patients [18,19,20]. These results suggest that MMSE scores are associated with plasma levels of AD-related biomarkers.

IMR, characterized by high sensitivity and specificity, has been applied to assay ultra-low concentration biomolecules, such as amyloid, tau protein, and α-synuclein in plasma. Several reports demonstrated the feasibility of quantifying Aβ_1-40_, Aβ_1-42_, tau, and α-synuclein in human plasma [21,22,23]. The levels of these biomarkers vary from tens of pg/mL to fg/mL. Clinical studies revealed that the power to discriminate between AD (or Parkinson’s disease) and normal controls is higher than 90% when using IMR [24]. Moreover, the relationships between plasma tau and hippocampal atrophy, as well as between plasma Aβ and amyloid positron emission tomography (PET), were clarified [25,26]. A recent study used IMR to measure plasma Aβ and tau levels in normal controls aged 23–91 years, enrolled at 11 sites in Taiwan, Japan, US, Sweden, and China [27]. These clinical data demonstrate the promise of IMR in clinical practice for the diagnosis of AD through plasma biomarker assays.

In the present study, we used an ultra-sensitive IMR assay to measure concentrations of plasma biomarkers, including Aβ_1-40_, Aβ_1-42_, t-Tau, and phosphorylated tau (Threonine 181) (p-Tau181). Using these data, we examined the correlations between MMSE scores and plasma levels of these biomarkers in patients with amnestic mild cognitive impairment (aMCI) or AD. This cross-sectional investigation included 90 participants at baseline; of them, five were followed up for 1–2 years to assess correlations between changes in MMSE scores and plasma biomarker levels.

## 2. Materials and Methods

### 2.1. Participant Recruitment

Between 1 January 2017 and 31 October 2018, 90 participants (13 healthy controls (HCs), 40 patients with aMCI, and 37 patients with AD) were recruited from the neurology outpatient clinic at the Tri-Service General Hospital (TSGH) of the National Defence Medical Centre, Taiwan. After a comprehensive medical examination, including a review of past medical problems, physical and neurological examinations, laboratory tests (creatinine, fasting blood sugar, free-thyroxine 4, high-sensitivity thyroid-stimulating hormone, vitamin B12, folic acid, and serologic tests for syphilis, white blood cells, red blood cells, haemoglobin, mean corpuscular volume, mean corpuscular haemoglobin, mean corpuscular haemoglobin concentration, and platelet count), and neuroimaging assessments, all participants were tested individually by the same clinical neuropsychologist at TSGH. The tests included the MMSE, the clinical dementia rating (CDR) scale, the short-form geriatric depression scale (GDS-S), memory tests (digit span and auditory verbal learning tests) [28], an executive function test (the Wisconsin card sorting test), an attention test (the symbol digit test), complex visuospatial perception tests (Rey complex figure test), the Wechsler adult intelligence scale-III, and the Taiwanese version of the Wechsler memory Scale, third edition [29]. Exclusion criteria were the presence of (i) specific uncontrolled medical conditions, including heart failure, sepsis, liver cirrhosis, renal failure, chronic obstructive pulmonary disease, poorly controlled diabetes (Hemoglobin A1c (HbA1C > 8.5)), recent myocardial infarction (within the past 6 months), or malignancy (during the past 2 years); (ii) substance abuse; (iii) medical history of stroke or Parkinson’s disease; and (iv) GDS-S scores > 9 or modified Rankin scale scores > 3. Participants were diagnosed as AD or aMCI by using the respective core criteria of the National Institute on Aging/Alzheimer’s Association [30,31]. For the diagnosis of aMCI, a formal cognitive examination was also applied, with a cut-off level at or below the fourth percentile (i.e., lower than 1.5 standard deviations) of the scale value for age- and education-matched controls. All healthy participants exhibited normal cognitive function, which was confirmed based on MMSE and CDR scores.

### 2.2. Ethical Approval and Consent to Participate

All volunteers or their main caregivers provided written informed consent prior to study enrollment. The study was approved by the institutional review board of the Tri-Service General Hospital (TSGHIRB 1-107-05-111).

### 2.3. Preparation of Plasma Samples

Non-fasting blood was drawn using 9 mL K3-EDTA tubes (455036, Greiner Bio-one GmbH, Kremsmünster, Austria), which were gently inverted three times immediately following blood collection. Blood samples were then centrifuged at a relative centrifugal force (RCF, 2300g) for 10 min (4 °C) by using a swing-out (bucket) rotor (5202R, Eppendorf). Each 0.4 mL plasma sample was transferred to a fresh 2.0 mL tube (CryzoTraq, Ziath, Cambridge, United Kingdom). All aliquoted plasma samples were stored at −80 °C within 8 h of blood collection until the IMR assay. Plasma samples were collected between 2017 and 2018, and IMR assays were performed in 2018.

### 2.4. Plasma Biomarker Assays

For each plasma sample, the levels of Aβ_1-40_, Aβ_1-42_, t-Tau, and p-Tau181 were assayed using IMR kits (MF-AB0-0060, MF-AB2-0060, MF-TAU-0060, and MF-PT1-0060, MagQu Co., New Taipei City, Taiwan). For each assay, 40 μL (Aβ_1-40_, T-Tau, and p-Tau181) or 60 μL (Aβ_1-42_) of plasma was mixed with 80 or 60 μL of reagent, respectively. Each reported biomarker concentration represents the average of duplicated measurements. An IMR analyzer (XacPro-S, MagQu Co., New Taipei City, Taiwan) was used for all assays. The reliable measurement ranges using IMR are from 0.17 to 1000 pg/mL for Aβ_1-40_, 0.77 to 30,000 pg/mL for Aβ_1-42_, 0.026 to 3000 pg/mL for t-Tau, and 0.0196 to 1000 pg/mL for p-Tau181. The intra-assay or inter-assay coefficient of variation for assaying Aβ_1-40_, Aβ_1-42_, t-Tau, or p-Tau181 using IMR is within the range of 7% to 10% for high-concentration quality control samples. For low-concentration quality control samples, the intra-assay or inter-assay coefficient of variation for assaying Aβ_1-40_, Aβ_1-42_, t-Tau, or p-Tau181 using IMR is within the range of 10% to 15%. For each kind of biomarker, two batches of reagent were used. The quality of each batch of reagents was well controlled by monitoring particle size, particle concentration, and bioactivity. The variation in these reagent properties between batches is lower than 10%.

### 2.5. Analysis of Apolipoprotein E Alleles

To efficiently obtain genetic information from samples collected from Taiwanese patients of Han Chinese ethnicity, the Taiwan Biobank (TWB) designed the TWB genotype array, based on the Affymetrix Axiom genotyping platform. The TWB genotype array enabled good-quality genotyping of 77 individuals. Two single-nucleotide polymorphisms (SNPs, rs429358 and rs7412) defining the apolipoprotein E (ApoE) isoforms were genotyped using the TWB array.

### 2.6. Statistical Analysis

Continuous variables are presented as mean ± standard deviation. Independent sample *t*-tests were used to compare continuous variables between HCs and patients with aMCI/AD. The relationships between MMSE scores and plasma biomarker levels at baseline and follow-up were assessed using Pearson’s correlation analysis. All tests were two-tailed, and the level of statistical significance was set at *p* < 0.05. No adjustments for multiple comparisons were made in this study. All analyses were performed using GraphPad Prism (GraphPad Software Inc., La Jolla, CA, USA).

## 3. Results

Baseline demographic information of the enrolled participants is presented in Table 1. The CDR scores were significantly higher in patients with aMCI (0.5 ± 1.0) and AD (1.5 ± 2.8) than in HCs. Furthermore, the MMSE scores, ranging from 6 to 30, were significantly lower in patients with aMCI (26.1 ± 2.8) and AD (20.2 ± 5.4) than in HCs (29.5 ± 0.5). Among the 90 enrolled participants, 54 underwent ApoE allele assessment, including six HCs, 16 aMCI patients, and 32 AD patients. The ApoE ε4 allele frequency ranged from 9.6% to 16.7% among the diagnostic groups. All plasma samples were detected within the limits of quantification of individual plasma biomarkers as assayed by IMR in the present study.

**Figure 1:**
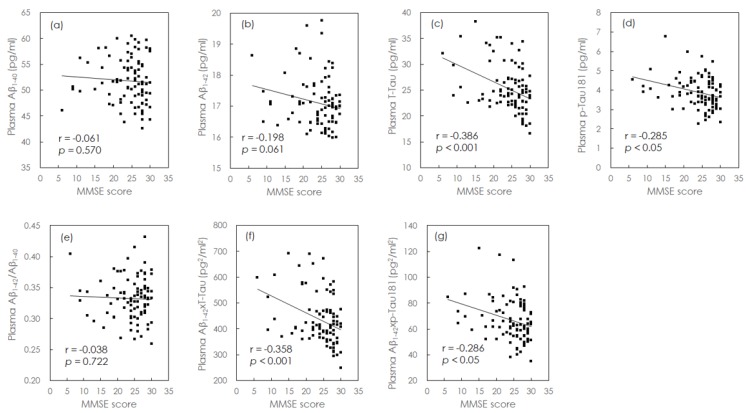
Individual and combined plasma biomarkers. Levels of (**a**) Aβ_1-40_, (**b**) Aβ_1-42_, (**c**) t-Tau (**d**) p-Tau181, (**e**) Aβ_1-42_/Aβ_1-40_, (**f**) Aβ_1-42_ × t-Tau, and (**g**) Aβ_1-42_ × p-Tau181 versus MMSE scores for the 90 participants (HCs, aMCI, and AD) in the cross-sectional analysis. Aβ: amyloid-β; MMSE: mini-mental state examination; HC: healthy control; aMCI: amnestic mild cognitive impairment; AD: Alzheimer’s disease; t-Tau: total tau; p-Tau181: phosphorylated tau 181.

We also investigated the relationships between plasma biomarkers and MMSE scores. Because the concentrations of plasma biomarkers investigated in the present study are independent of age and education levels [22,32], our analyses focused on unadjusted MMSE scores. Individual plasma biomarker levels are plotted against MMSE scores in Figure 1.

The solid grey line represents the Pearson correlation. Although there was no association between plasma Aβ_1-40_ levels and MMSE scores (Figure 1a), plasma Aβ_1-42_ showed a trend, and p-Tau181 levels exhibited a weak negative correlation with MMSE scores (Aβ_1-42_: r = −0.198, *p* = 0.061; p-Tau181: r = −0.285, *p* < 0.05), as shown in Figure 1b and 1d. In addition, we observed a moderate negative correlation between plasma levels of t-Tau and MMSE scores (r = −0.386, *p* < 0.001; Figure 1c). Given that the plasma levels of t-Tau and p-Tau181 were negatively correlated with MMSE scores, our results suggest that increases in the levels of these biomarkers correspond to more severe cognitive impairment.

We then investigated the cross-sectional relationships between combinations of plasma biomarkers and MMSE scores (Figure 1). Although we observed no significant correlation between plasma Aβ_1-42_/Aβ_1-40_ levels and MMSE scores (Figure 1e), Aβ_1-42_ × t-Tau plasma levels exhibited a moderate negative correlation with MMSE scores (r = −0.358, *p* < 0.001). In addition, Aβ_1-42_ × p-Tau181 levels exhibited a weak negative correlation with MMSE scores (r = −0.286, *p* < 0.05; Figure 1e,f). Plasma levels of t-Tau were most strongly correlated with MMSE scores (Figure 1), suggesting that plasma t-Tau levels are more closely associated with cognitive impairment than Aβ_1-42_ or p-Tau levels.

**Figure 2:**
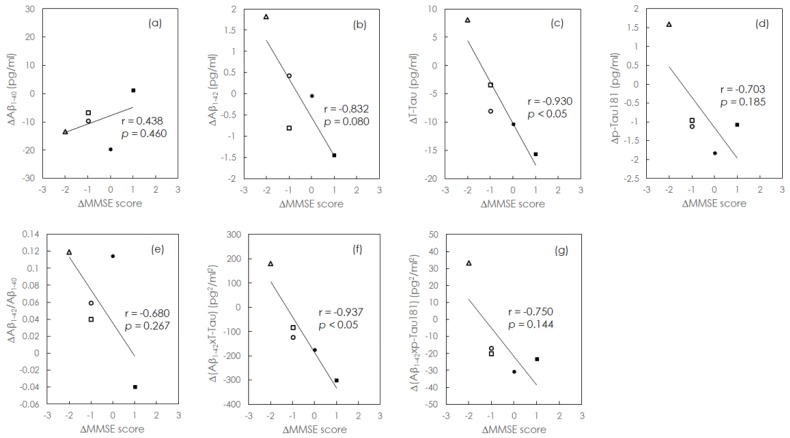
Changes in individual and combined plasma biomarkers for two AD patients (■ and •) and three aMCI patients (○, □, and △). Levels of (**a**) Aβ_1-40_, (**b**) Aβ_1-42_, (**c**) t-Tau, (**d**) p-Tau181, (**e**) Aβ_1-42_/Aβ_1-40_, (**f**) Aβ_1-42_ × t-Tau, and (**g**) Aβ_1-42_ × p-Tau181 versus changes in MMSE scores for the five individual patients with aMCI (○, □, and △) or AD (■ and •) in the longitudinal analysis.

Among the patients included at baseline, two with AD and three with aMCI were followed up for 1–2 years. Demographic information of these five patients is presented in Table 2. The MMSE scores and plasma levels of Aβ_1-40_, Aβ_1-42_, t-Tau, and p-Tau181 were examined at each follow-up visit. The change in MMSE score for a given patient was defined as follows: ΔMMSE score = follow-up MMSE score—baseline MMSE score. Similarly, changes in biomarker levels were defined as follows: Δbiomarker = follow-up biomarker concentration – baseline biomarker concentration. The MMSE scores decreased (ΔMMSE score < 0) in three patients, improved (ΔMMSE score > 0) in one patient, and remained stable (ΔMMSE score = 0) in one patient. The corresponding changes in plasma biomarker levels are plotted in Figure 2a–g. As shown in Figure 2a, plasma Aβ_1-40_ levels showed a decreasing trend in patients exhibiting decrease of cognitive function (r = 0.438, *p* = 0.46). However, plasma levels of Aβ_1-42_, t-Tau, and p-Tau181 showed an increasing trend in patients exhibiting a decrease in cognitive function (Aβ_1-42_: r = −0.832, *p* = 0.08; t-Tau: r = −0.932, *p* < 0.05; p-Tau181: r = −0.703, *p* = 0.185; Figure 2b–d).

The relationships between changes in combinations of plasma biomarkers and changes in MMSE scores during the follow-up period are shown in Figure 2e–g. Figure 2e indicates that greater changes in plasma Aβ_1-42_/Aβ_1-40_ levels were associated, at the trend level, with more severe decreases in cognitive function (r = −0.680, *p* = 0.267). Figure 2f,g indicates that plasma levels of both Aβ_1-42_ × t-Tau and Aβ_1-42_ × p-Tau181 increased, at the trend level, with decreases in cognitive function (r = −0.937, *p* < 0.05 and r = −0.750, *p* = 0.144, respectively). These results suggest that individual levels of plasma biomarkers are correlated with the degree of cognitive decline. In particular, strong correlations were observed between increases in the concentration of plasma t-Tau and cognitive decline.

## 4. Discussion

IMR revealed that t-Tau and p-Tau plasma concentrations were correlated with MMSE scores in aMCI and AD patients. Longitudinal analyses indicated that increases in plasma concentrations of t-Tau were strongly associated with cognitive decline.

Aβ plaques in the brain are pathological hallmarks of AD. In addition to neuropathological analyses and neuroimaging, studies showed that levels of Aβ_1-40_ or Aβ_1-42_ in biofluids can be used to assess the formation of amyloid plaques in the brain [8,12]. PET showed that plasma Aβ_1-42_/Aβ_1-40_ levels are moderately correlated with the area of Aβ plaques in the frontal lobe, parietal lobe, temporal lobe, and precuneus [25]. A high accumulation of Aβ can increase toxicity in the brain and may lead to severe impairment or decline in cognition [4,6].

Instead of a sandwich technology involving two antibodies against the target biomolecule, IMR uses only one antibody [33] and nanoparticles of biofunctionalized antibodies as substrates, which are homogeneously dispersed in the reagent, so that the binding area for assaying biomolecules is extremely high, leading to high sensitivity. The spin-wash technology is applied to IMR to significantly suppress the matrix effect or non-specific bindings [34]. IMR is therefore wash-free and dilution-free, and thus very user-friendly. Additionally, the signal detected by IMR is magnetic, rather than optical, so that the interference by sample color due to hemoglobin or bilirubin is eliminated. Thus, high specificity is achieved with IMR. Given the high sensitivity and specificity, there is no need to perform pre-purification or extraction of the target biomolecules before the measurements. The plasma samples after centrifugation can instead be directly used for IMR measurements.

We observed no significant correlations between the cognitive impairment severity and plasma Aβ_1-40_ or Aβ_1-42_/Aβ_1-40_ levels (Figure 1a,e); however, plasma Aβ_1-42_ levels exhibited a trend toward negative correlation with the severity of cognitive impairment (Figure 1b), while decreases in plasma Aβ_1-40_ and increases in plasma Aβ_1-42_/Aβ_1-40_ were both associated with a trend toward cognitive decline (Figure 2a,e). We also observed a trend-level negative correlation between increases in plasma Aβ_1-42_ levels and cognitive decline (Figure 2b). Thus, our results indicate that although plasma levels of Aβ_1-40_ and Aβ_1-42_, and their ratio may not aid in determining the severity of cognitive impairment, their longitudinal changes may be useful for monitoring cognitive decline. Studies have reported significant, though inconsistent, effects of treatment on plasma Aβ levels. In the Alzheimer’s Disease Cooperative Study (ADCS) MCI trial, patients in the donepezil and vitamin E groups exhibited decreased plasma Aβ levels at the 3-year follow-up. However, in the ADCS simvastatin trial, patients in the simvastatin group exhibited increased plasma Aβ levels at the 18-month follow-up. Presumptive symptomatic agents such as donepezil and vitamin E appear to influence plasma levels of Aβ in patients with AD, warranting further trials on the impact of medical treatment on blood-based biomarkers of MCI/AD [35].

The accumulation of tau protein tangles in the brain represents another pathological hallmark of AD. t-Tau primarily functions to stabilize axonal microtubules. In patients with AD, the tau protein is abundantly expressed in the brain, leading to changes in the concentration of t-Tau in the CSF and plasma. Such changes are typically accompanied by neuronal damage and brain atrophy in the later stages of the disease [36]. Studies have indicated that decreases in grey matter, hippocampal, and amygdala volumes contribute to increased t-Tau plasma levels. Brain atrophy is associated with cognitive dysfunction, hence plasma t-Tau concentrations increase with the progression of cognitive decline [9], as demonstrated in Figure 2c,f. Moreover, in our study, the concentration of plasma t-Tau exhibited a moderate negative correlation with the severity of cognitive impairment (Figure 1c). These results suggest that both the baseline concentration and longitudinal changes of t-Tau are related to the severity of cognitive impairment or decline.

Biologically, hyperphosphorylation of the tau protein can directly lead to neuronal or axonal death in patients with AD. Thus, p-Tau is also considered a potential biomarker for the early detection of AD [37]. Studies have demonstrated that CSF levels of p-Tau are significantly higher in patients with AD than in HCs. CSF levels of p-Tau can also be used to discriminate AD from other forms of dementia [38,39,40,41]. Assaying the CSF levels of p-Tau can increase the diagnostic accuracy of AD by improving sensitivity and specificity. Furthermore, recent developments have facilitated the evaluation of p-Tau levels in blood samples, suggesting that plasma p-Tau levels can be used for monitoring neurodegeneration in AD patients. However, p-Tau levels are considerably lower in the plasma than in the CSF [9,42], thus requiring an extremely sensitive assay rather than the current ELISA methods. Recent studies have documented the feasibility of IMR for the detection of plasma p-Tau181, which is present at very low concentrations. Indeed, one study noted that p-Tau181 levels could be used to categorize disease severity in patients with early-stage AD [32].

**Figure 3:**
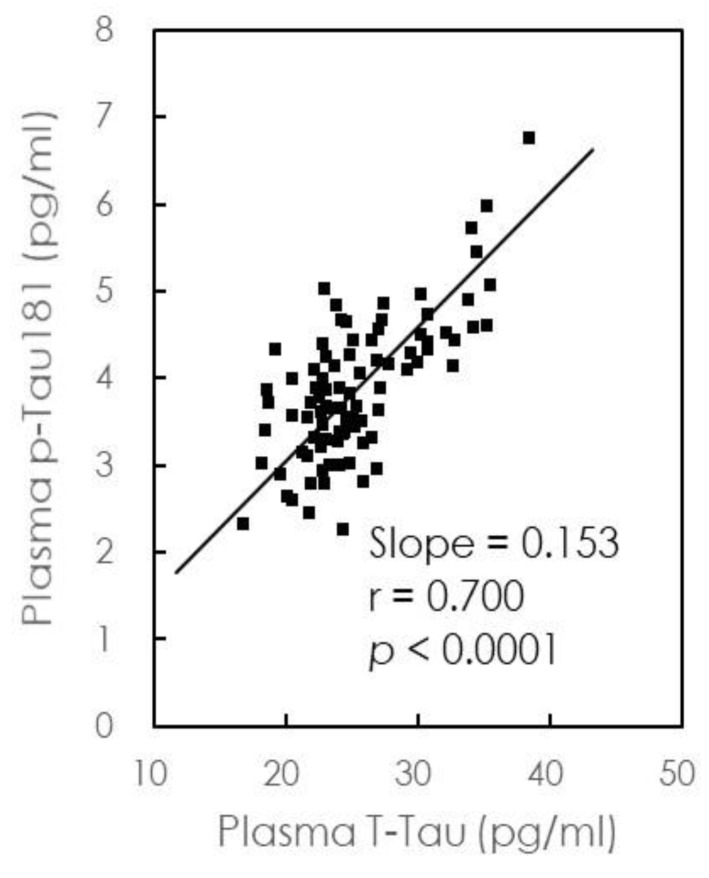
Relationship between plasma levels of p-Tau181 and t-Tau for all participants in the cross-sectional study.

When plasma levels of p-Tau181 were plotted against t-Tau levels (Figure 3), we observed a concentration of p-Tau approximately equal to 15% of that of t-Tau, in agreement with a previous study [32], suggesting that the relative concentrations of p-Tau181 and t-Tau are similar in HCs and AD patients. Similar trends can be observed in Figure 1c,d,f,g and Figure 2c,d,f,g. However, further analyses revealed that t-Tau levels were more strongly correlated with MMSE scores and cognitive decline than p-Tau181 levels.

In this study, the plasma t-Tau and p-Tau181 levels were elevated in AD as compared to those in HCs. The elevations were consistent with that observed for CSF t-Tau. However, the plasma Aβ_1-42_ level increased in AD, in contrast to the decrease in CSF Aβ_1-42_ seen in AD. These opposing changes compared to HCs between plasma Aβ_1-42_ (assayed with IMR) and CSF Aβ_1-42_ (assayed with ELISA) depend on the assay methodology used. Different assay technologies involve different procedures for plasma preparation and antibody use, suggesting that different conformations of Aβ_1-42_ in plasma are detected by different assay technologies. In the study by Teunissen et al., a negative correlation was found between plasma Aβ_1-42_ and CSF Aβ_1-42_, thus, demonstrating the opposing changes in plasma Aβ_1-42_ detected with IMR as compared to the changes in CSF Aβ_1-42_ assayed with ELISA [43]. Therefore, caution is recommended when comparing findings among different studies.

Previous studies have investigated the potential use of blood biomarkers for disease severity in the Taiwanese population with AD using ELISA. The authors reported a correlation between the MMSE score and total Aβ levels, instead of t-Tau and p-Tau levels, in the AD group [44], a finding which differs from our findings. The use of differing bioanalytical methods with varied assay capacities of Aβ aggregations or Aβ bound to other proteins may partially explain the discrepancy in results between the above studies. The IMR assay utilized in the present study demonstrated better consistency and sensitivity in assaying quite low levels of plasma Aβ than conventional ELISA. Other studies [18] have explored blood-based biomarkers in Taiwanese patients with MCI/AD by IMR and concluded that plasma Aβ_1-42_ is a useful biomarker for AD. The use of a relatively large sample size and longer follow-up duration in the current study helped to validate the clinical use of these plasma biomarkers.

In accordance with previous IMR studies, our results support the notion that plasma levels of biomarkers including t-Tau and p-Tau181 can be used to differentiate HCs and AD patients in both the prodromal and dementia phases. However, in this study, no single biomarker or combination of biomarkers was able to distinguish aMCI from AD, in contrast to previous findings suggesting that the combination of Aβ_1-42_ and tau protein could be used to differentiate AD in the prodromal and dementia phases [22] and that plasma levels of p-Tau181 could be used to categorize disease severity in patients with early-stage AD [32]. Heterogeneity in sample preparation methods, study designs, and outcome measurements may explain this discrepancy. Therefore, standardizing the sample-handling methods is necessary to minimize interactions with other blood components and collection materials. Future studies should also aim to control for confounding factors such as therapeutic effects to enhance the reproducibility of the results.

Among the five follow-up cases, one had cognitive improvement with an overall increase in MMSE score by one point during the 2-year follow-up period. Although AD is a neurodegenerative disease characterized by progressive cognitive decline, several studies have observed that exercise intervention might improve cognitive function in people with AD [45]. These findings indicate that cognitive improvement in AD still might be noted in short follow-up periods.

Our longitudinal analyses indicated that plasma concentrations of t-Tau were strongly correlated with cognitive decline in patients with aMCI/AD. Recent multicenter Alzheimer’s disease neuroimaging initiative studies in North America have revealed that plasma levels of neurofilament light (NfL) are increased at baseline in MCI and AD patients [46]. Moreover, in accordance with our preliminary findings, NfL levels increased at greater rates in patients with preclinical AD, prodromal AD, and AD dementia than in HCs. Therefore, longitudinal t-Tau levels may be useful for tracking the dynamic evolution of neurodegeneration throughout the progression of AD. There are currently no non-invasive, blood-based methods for objectively monitoring the AD neurodegenerative process, hence, our findings have significant clinical implications.

To the best of our knowledge, this is the first longitudinal study to assess correlations between changes in MMSE scores and plasma biomarker levels in patients with aMCI/AD by IMR. However, several limitations must be considered when interpreting our findings. First, our study cohorts were restricted to clinically assessed patients with cognitive test results, and we did not obtain CSF samples, or amyloid or tau PET scans, to investigate the correlations between changes in cognition and other biomarkers (e.g., CSF markers and imaging). Due to cultural traditions in Taiwan, collecting CSF samples from patients with aMCI/AD and HCs is difficult. The clinical and pathological findings for AD vary widely, and the wide ranges of plasma concentration of Aβ_1-42_ and tau protein are consistent with these findings. The plasma Aβ_1-42_ and Tau-protein concentrations quantified by IMR still allow differentiation between healthy subjects and aMCI/AD patients based on the results of previous studies [22]. Second, due to limited funds and a relatively short study period, only five patients were enrolled in the follow-up analysis of the present study. The relatively small sample size and short follow-up period may limit statistical analysis results. Accordingly, we intend to increase the follow-up sample size in our next study. Further long-term studies involving larger numbers of participants are required and are expected to validate the preliminary results of our longitudinal study. Third, owing to limitation of funds, a few plasma samples collected in early 2017 could not be analyzed promptly, which might have potentially produced biased results. However, all plasma samples were stored at −80 °C within 8 h of blood collection, which complied with the standard operating procedures for plasma collection. Fourth, previous studies have shown that t-Tau can be assayed in numerous peripheral organs, such as the submandibular gland, abdominal skin, scalp, colon, and liver in either AD or non-AD subjects [47]; these studies have also documented the decreased level of certain tau species, p-Tau231, in AD submandibular glands, which was inversely correlated with the Braak neurofibrillary tangle stage. Hence, potential systemic bias might exist while using these peripheral measurements.

In conclusion, our cross-sectional IMR analyses revealed that plasma concentrations of t-Tau and p-Tau were correlated with MMSE scores in patients with aMCI and AD. Our longitudinal study revealed that increases in plasma concentrations of t-Tau were strongly correlated with cognitive decline. These findings suggest that plasma levels of t-Tau can be used to assess the severity of cognitive impairment, and their changes to monitor cognitive decline in patients with aMCI/AD.

## Figures and Tables

**Table 1:** Baseline demographic information of enrolled participants.

Diagnostic Group	HC	Patients	Patients	
			aMCI	AD
N (female %)	13 (76.9%)	77 (77.9%)	40 (80%)	37 (75.7%)
Age (years)	64.4 ± 5.7	74.6 ± 8.2 **	72.4 ± 7.6	77.0 ± 8.3
Education (years)	11.2 ± 3.8	8.9 ± 4.9 *	8.0 ± 4.5	9.8 ± 5.2
CDR	0.0 ± 0.0	1.1 ± 2.0	0.5 ± 1.0	1.5 ± 2.8
MMSE	29.5 ± 0.5	23.2 ± 5.2 **	26.1 ± 2.8	20.2 ± 5.4
ApoE ε4 allele frequency (N)	16.7% (6)	9.8% (48)	9.6% (16)	15.6% (32)
Aβ_1-40_ (pg/mL)	51.8 ± 5.1	51.8 ± 4.3	51.9 ± 4.9	51.7 ± 3.7
Aβ_1-42_ (pg/mL)	16.7 ± 0.7	17.2 ± 0.8 *	17.0 ± 0.7	17.4 ± 1.0
t-Tau (pg/mL)	22.5 ± 3.4	25.8 ± 4.5 *	24.5 ± 4.0	27.1 ± 4.8
p-Tau181 (pg/mL)	3.53 ± 0.55	3.95 ± 0.83 *	3.82 ± 0.71	4.09 ± 0.94
Aβ_1-42_/Aβ_1-40_	0.326 ± 0.035	0.330 ± 0.034	0.330 ± 0.035	0.338 ± 0.032
Aβ_1-42_ × t-Tau (pg^2^/mL^2^)	377.8 ± 65.8	441.7 ± 97.6 *	418.3 ± 80.3	473.2 ± 107.4
Aβ_1-42_ × p-Tau181 (pg^2^/mL^2^)	59.1 ± 10.3	68.2 ± 16.7 *	65.0 ± 13.3	71.6 ± 19.4

CDR: clinical dementia rating; MMSE: mini-mental state examination; Aβ: amyloid-β; t-Tau: total tau; p-Tau: phosphorylated tau 181; HC; healthy control; aMCI: amnestic mild cognitive impairment; AD: Alzheimer’s disease; ApoE: apolipoprotein E; * *p* < 0.05 compared with the HC group; ** *p* < 0.001 compared with the HC group.

**Table 2:** Demographic information of the five patients with aMCI/AD enrolled in the longitudinal study.

Patient	A		B		C		D		E	
Baseline diagnostic group	aMCI		aMCI		aMCI		AD		AD	
Gender	Female		Female		Female		Male		Female	
Visit	B	F	B	F	B	F	B	F	B	F
Age (years)	72	74	80	82	87	88	95	97	73	74
MMSE	25	24	28	27	27	25	20	21	21	21
Aβ_1-40_ (pg/mL)	60.5	50.8	49.0	42.3	59.2	45.7	47.0	48.3	64.1	44.4
Aβ_1-42_ (pg/mL)	16.1	16.6	18.2	17.4	16.0	17.9	17.7	16.3	16.8	16.7
t-Tau (pg/mL)	24.3	16.2	30.1	25.8	20.4	28.5	32.6	16.9	27.8	17.5
p-Tau181 (pg/mL)	3.38	2.27	4.50	3.55	2.62	4.21	4.16	3.10	4.25	2.43
Aβ_1-42_/Aβ_1-40_	0.267	0.326	0.372	0.412	0.271	0.390	0.376	0.337	0.262	0.377
Aβ_1-42_ × t-Tau (pg^2^/mL^2^)	391.4	268.5	549.5	466.3	326.7	508.4	576.1	274.5	466.9	292.5
Aβ_1-42_ × p-Tau181 (pg^2^/mL^2^)	54.5	37.6	82.1	62.0	42.0	75.2	73.7	50.4	71.3	40.6

MMSE: mini-mental state examination; Aβ: amyloid-β; t-Tau: total tau; p-Tau181: phosphorylated tau 181; B: baseline visit; F: follow-up visit.

## Data Availability

The dataset generated and analyzed in the current study is available from the corresponding author on reasonable request.

## Abbreviations

CDR = clinical dementia rating; MMSE = mini-mental state examination; Aβ = amyloid-β; t-Tau = total tau; p-Tau181 = phosphorylated tau 181; HC = healthy control; aMCI = amnestic mild cognitive impairment; AD = Alzheimer’s disease; IMR = immunomagnetic reduction; CSF = cerebrospinal fluid; PET = positron emission tomography.
